# Supplementary material for: iPSCs in Neurodegenerative Disorders: A Unique Platform for Clinical Research and Personalized Medicine
Source: J Pers Med. 2022 Sep 10;12(9):1485. doi: 10.3390/jpm12091485 (PMC9500601; doi:10.3390/jpm12091485)
Supplement: Supplementary file 1 [file jpm-12-01485-s001.zip › Table-1.pdf]

Supplementary Table S1: Case Report published for Parkinson's disease in PUBMED

S. No.	Article Type	Journal	Author	Topic	Year	Cell line	Gender	Age	Reprogramming	Source	Disease
1	Case Report	Stem Cell Res.	Chen ZT et al.	Generation of an induced pluripotent stem cell line, FJMUUHi001-A, from a hereditary Parkinson's disease patient with homozygous mutation of c.189dupA in PARK7	2021	FJMUUHi001-A	Male	32	Electrotransfection with 4 episomal vectors with 5-reprogramming-factor genes (OCT3/4, SOX2, c-MYC, KLF4, and BCL-XL)	PBMCs	Parkinson's disease
2		N Engl J Med.	Schweitzer JS et al.	Personalized iPSC-Derived Dopamine Progenitor Cells for Parkinson's Disease.	2020	C4-mDAPs, H9-mDAPs	Male	69	Direct delivery of Oct4, Sox2, Klf4, and c-Myc fused with cell penetrating peptide	Skin fibroblasts	Idiopathic Parkinson's disease
3		Stem Cell Res.	Larsen SB et al.	Induced pluripotent stem cell line (LCSBi001-A) derived from a patient with Parkinson's disease carrying the p.D620N mutation in VPS35	2020	LCSBi001-A	Male	76	CytoTune™-iPS 2.0 Sendai Reprogramming Kit	Skin fibroblasts	VPS35 p.D620N mutation associated Parkinson's disease
4		Stem Cell Res.	Chen M et al.	Generation of an induced pluripotent stem cell line (DANi-011A) from a Parkinson's disease patient with a LRRK2 p.G2019S mutation	2020	DANi-011A	Female	70	Non-integrating Sendai virus with POU5F1, SOX2, KLF4 and MYCL	Skin fibroblasts	Parkinson's disease 8
5		Stem Cell Res.	Azevedo C et al.	Generation of an induced pluripotent stem cell line (CSC-32) from a patient with Parkinson's disease carrying a heterozygous variation p.A53T in the SNCA gene	2020	CSC-32	Male	45	Non-integrating Sendai virus with OCT3/4, SOX2, c-MYC and KLF4	Skin fibroblasts	SNCA mutation associated Parkinson's disease
6		Acta Neuropathol Commun.	Chumarina M et al.	Cellular alterations identified in pluripotent stem cell-derived midbrain spheroids generated from a female patient with progressive external ophthalmoplegia and parkinsonism who carries a novel variation (p.Q811R) in the POLG1 gene	2019	CSC-35E, CSC-35P, CSC-35 V	Female	38	Sendai virus delivery of OCT3/4, KLF-4, SOX-2 and c-MYC	Skin fibroblasts	POLG1 mutation associated early onset Parkinson's disease
7		Bull Exp Biol Med.	Vetchinova AS et al.	Cytogenetic Analysis of the Results of Genome Editing on the Cell Model of Parkinson's Disease	2018	N/A Not specificied	Male	N/A Not given	Vectors and single-stranded donor DNA Molecules delivered by Lipophilic transfection (TurboFect, Thermo Fisher Scientific)	Skin fibroblasts	Parkinson's disease 8
8		Stem Cell Res.	Lee SY	Generation of gene-corrected iPSC line from Parkinson's disease patient iPSC line with alpha-SNCA A53T mutation	2018	KIOMi001-A	Female	51	Episomal plasmids (pCXLE-hOCT3/4-shp53, pCXLE-hU, pCXLE-hSK)	Skin fibroblasts	α-SNCA mutation associated Parkinson's disease
9		Stem Cell Res.	Wang Y et al.	Generation of induced pluripotent stem cell line (ZZUi005-A) from a 21-year-old patient with a novel RAB39B gene mutation in X-linked juvenile parkinsonism	2017	ZZUi005-A	Male	21	Sendai virus	Skin fibroblasts	RAB39B mutation associated X-linked Parkinson's disease

10		Parkinsonism Relat Disord.	Mishima T et al.	Cytoplasmic aggregates of dynactin in iPSC-derived tyrosine hydroxylase-positive neurons from a patient with Perry syndrome	2016	N/A	Male	61	Episomal vectors carrying OCT3/4, SOX2, KLF4, L-MYC, LIN28, EBNA1, and p53 carboxy-terminal dominant-negative fragment	PBMCs	Perry syndrome
11		PLoS One	Byers B et al.	SNCA triplication Parkinson's patient's iPSC-derived DA neurons accumulate α-synuclein and are susceptible to oxidative stress	2011	N/A	Male	48	Retroviral gene instertion of OCT4, SOX2, KLF4 and c-MYC	Skin fibroblasts	α-SNCA mutation associated Parkinson’s disease
