# Supplementary material for: iPSCs in Neurodegenerative Disorders: A Unique Platform for Clinical Research and Personalized Medicine
Source: J Pers Med. 2022 Sep 10;12(9):1485. doi: 10.3390/jpm12091485 (PMC9500601; doi:10.3390/jpm12091485)
Supplement: Supplementary file 1 [file jpm-12-01485-s001.zip › Table-2.pdf]

Supplementary Table S2: Case Report published for Alzheimer's disease in PUBMED

S. No.	Article Type	Journal	Author	Topic	Year	Cell line	Gender	Age	Reprogramming	Source	Disease
1	**Case Report**	Stem Cell Res	Wang J et al.	Induced pluripotent stem cells derived from one 70-years-old male donor with the APOE-ε4/ε4 alleles	2021	HEBHMUi008-A	Male	70	Sendai virus mediated delivery of OCT3/4, SOX2, cMYC and KLF4	PBMC	Cerebral artery insufficiency
2		Stem Cell Res.	Zhang L et al.	Generation of induced pluripotent stem cell line (IPTi002-A) from an 87-year old sporadic Alzheimer's disease patient with APOE3 (ε3/ε3) genotype	2021	IPTi002-A	Female	85	Transgene free, Episomal plasmid	PBMC	AD
3		Stem Cell Res.	Wang Z et al.	Generation of induced pluripotent stem cell line (ZZUi0024-A) from a 51-year-old patient with APP gene mutation in Alzheimer's disease	2021	ZZUi0024-A	Female	51	Electroporated with episomal plasmids	Skin fibroblasts	AD
4		Stem Cell Res	Dai Q et al.	Generation of an induced pluripotent stem cell line (SIAISi003-A) from a 79-year-old patient with Alzheimer's disease having APOE3/4 genetic background	2020	SIAISi003-A	Female	79	Transgene free, episomal plasmids	PBMC	AD
5		Cell Prolif.	Li L et al.	Pathological manifestation of the induced pluripotent stem cell-derived cortical neurons from an early-onset Alzheimer's disease patient carrying a presenilin-1 mutation (S170F)	2020	PS1-S170F	Male	33	Sendai virus vector which expresses four reprogramming factors (OCT3/4, SOX2, cMYC and KLF4	PBMC	AD
6		Stem Cell Res.	Yan YI et al.	Establishment of SIAISi001-A, an induced pluripotent stem cell (iPSC) line from 66-year old mild cognitive impairment (MCI) with two copies of APOE4 gene	2020	SIAISi001-A	Female	66	Non-integrating Sendai virus mediated reprogramming	PBMCs	Mild cognitive impairment (MCI)
7		Stem Cell Res.	Wang Y et al.	Establishment of TUSMi007-A, an induced pluripotent stem cell (iPSC) line from an 83-year old Chinese Han patient with Alzheimer's disease (AD)	2018	TUSMi007-A	Male	83	Transgene free, Episomal plasmids	PBMCs	AD
8		Stem Cell Res	Muñoz SS et al.	Generation and characterization of human induced pluripotent stem cell lines from a familial Alzheimer's disease PSEN1 A246E patient and a non-demented family member bearing wild-type PSEN1	2018	UOWi002-A	Female	75	Transgene free	Dermal fibroblast	Non-demented control
						UOWi003-A		56			Familial AD
9		Stem Cell Res.	Wang Y et al	Derivation of induced pluripotent stem cells TUSMi006 from an 87-year old Chinese Han Alzheimer's disease patient carrying GRINB and SORL1 mutations	2018	TUSMi006-A	Male	87	Transgene free, Episomal plasmids	PBMCs	AD

10		Stem Cell Res.	Martins S et al	Lymphoblast-derived integration-free iPSC line AD-TREM2-3 from a 74 year-old Alzheimer's disease patient expressing the TREM2 p.R47H variant	2018	HHUUKDi004-A	Male	74	Episomal vectors oriP/EBNA-1 backbone containing OCT4, SOX2, KLF4, LIN28, LMYC and a p53 shRNA	Human lymphoblast cell	AD
11		Stem Cell Res.	Lehtonen Š et al	Generation of a human induced pluripotent stem cell line from a patient with a rare A673T variant in amyloid precursor protein gene that reduces the risk for Alzheimer's disease	2018	UEFi001-A	Male	65	Sendai virus delivery of OCT-3/4, KLF-4, SOX-2 and c-MYC genes	Skin fibroblasts	Protective against AD
12		Stem Cell Res.	Martins S et al	Lymphoblast-derived integration-free iPSC line AD-TREM2-1 from a 67year-old Alzheimer's disease patient expressing the TREM2 p.R47H variant	2018	HHUUKDi003-A	Male	67	Episomal vectors oriP/EBNA-1 backbone containing OCT4, SOX2, KLF4, LIN28 and L-MYC	Human lymphoblast cells	AD
13		Stem Cell Res.	Wang Z et al	Establishment of induced pluripotent stem cell line (ZZUi010-A) from an Alzheimer's disease patient carrying an APP gene mutation	2017	ZZUi010-A	Female	52	Sendai virus	Fibroblasts	AD
