# Supplementary material for: iPSCs in Neurodegenerative Disorders: A Unique Platform for Clinical Research and Personalized Medicine
Source: J Pers Med. 2022 Sep 10;12(9):1485. doi: 10.3390/jpm12091485 (PMC9500601; doi:10.3390/jpm12091485)
Supplement: Supplementary file 1 [file jpm-12-01485-s001.zip › Table-3.pdf]

Supplementary Table S3: Clinical Application of induced pluripotent stem cells (iPSCs) in Neurologic Disorders.

S. No.	Study title	Identifier	Intervention	Involvement of iPSCs	Status	Start date	Completion date	Participants	Country
1	Creation of a Bank of Fibroblast from Patients with Amyotrophic Lateral Sclerosis: Pilot Study (ALSCELL)	NCT01639391	Skin biopsy	Creation of a repository of fibroblasts from patients with ALS for later induction of iPSCs	Completed	November 2012	March 2015	19	France
2	Understanding Clinical Phenotype and Collecting Biomarker Samples in C9ORF72 ALS	NCT02686268	Blood samples	Induction of iPSCs from patients with C9ORF72 ALS as secondary objective		February 2015	October 2018	128	USA
3	Development of the Tool " iPSC " for the Functional Study of Mutations Responsible for Mental Retardation (Rementips)	NCT02980302	Skin biopsy	Creation of iPSC from human fibroblast with MYT1L mutation		September 2015	September 2017	4	France
4	Imaging and BioFluid Biomarkers in Amyotrophic Lateral Sclerosis (TRACK-ALS)	NCT02559869	Blood samples	Identification of imaging and biofluid biomarkers in people with ALS as secondary objective		November 2015	June 2019	86	USA
5	Answer ALS: Individualized Initiative for ALS Discovery (AnswerALS)	NCT02574390	Blood samples	Creation repository of iPSCs for ALS gene identification		December 2015	January 2020	1049	USA
6	Induced Pluripotent Stem Cells for Niemann Pick Disease (IPSNPABC)	NCT03883750	Skin biopsy	Induction of iPSCs from patients with Niemann-Pick disease with later differentiation into neural cells to study the misfolded proteins in endoplasmic reticulum		June 2018	December 2019	40	Pakistan
7	Establishment of Human Cellular Disease Models for Wilson Disease (IPSWILSON)	NCT03867526	Skin biopsy	Establishment of human cellular disease models for Wilson disease		June 2018	December 2019	40	Pakistan
8	Studies of Autistic Patients: Gene Networks and Clinical Subtypes	NCT01092208	Skin biopsy, blood samples	Induction of iPSCs from patients with Autism and/or Tuberous Sclerosis	Terminated	March 2010	September 2013	11	USA
9	Cell-Based Approaches for Modeling and Treating Ataxia-Telangiectasia	NCT02246491	Skin biopsy, blood samples	Induction of iPSCs from patients with Ataxia-Teleangiectasia		February 2015	July 2018	6	USA
10	Stem Cells in NF1 Patients with Tumors of the Central Nervous System	NCT03332030	Blood samples	Establish an iPSCs bank for phenotypically well-characterized patients with neurofibromatosis type I	Suspended (due to cessation of funding)	November 2015	July 2025	20	USA
11	Establishing of Neuronal-like Cells from Patients with Cisplatin-Induced Peripheral Neuropathy	NCT02492360	Blood samples	Samples taken for future creation of iPSC to create a model for cisplatin-induced peripheral neuropathy	Withdrawn	December 2017	December 2019	0	USA
12	Generation of a Cellular Model of CADASIL From Skin Fibroblasts	NCT02032225	Skin biopsy	Induction of iPSCs from patients with CADASIL for later differentiation into vascular smooth muscle cells	Unknown	February 2014	August 2018	10	France
13	Painful Channelopathies Study (PCS)	NCT02696746	Skin biopsy	Creation of iPSCs from patients with heritable pain syndromes as secondary objective		February 2012	June 2021	100	United Kingdom
14	Multi-Omics and IPSCs to Improve Diagnosis of Rare Intellectual Disabilities (MIDRID)	NCT03635294	Blood samples	Derivation of iPSC-derived neural progenitors from patients with intellectual disability for whole genome sequencing		January 2019	March 2020	6	France

15	Establishment of Genetic Basis for Neurological Disease by Genetic Screening	NCT03322306	Skin biopsy	Establishment of disease-specific iPSCs repositories from hereditary neurological diseases		November 2012	December 2025	200	Hong Kong
16	Derivation of Human Induced Pluripotent Stem (iPS) Cells to Heritable Cardiac Arrhythmias	NCT02413450	Skin biopsy, blood samples	Induction of iPSCs from patients with hereditary cardiac arrythmias for later differentiation into cardiomyocytes		August 2013	August 2025	100	USA
17	Study of Neurodegenerative Diseases Induced Stem Cells in Patients and Healthy Family Controls. (NeuronsiPS)	NCT03682458	Blood samples	Induction of iPSCs for later differentiaton into neurons and glia for the study of the pathological mechanisms underlying neuropathology		October 2019	October 2022	50	Italy
18	Comprehensive Analysis Platform To Understand, Remedy and Eliminate ALS (CAPTURE ALS)	NCT05204017	Blood samples	Creation of a repostory of iPSCs from patients with ALS as secondary objective		January 2022	August 2023	150	Canada
19	Biometric and Biological Data for Diagnosis and Therapy of Pain Patients (Bio2Treat)	NCT04835779	Blood samples	Induction of iPSCs from patients with small fiber neuropathy for later differentiation into sensory neurons as secondary objective		October 2010	October 2023	48	Germany
20	Prospective Evaluation of Infants With Spinal Muscular Atrophy: (SPOTSMA)	NCT02831296	Skin biopsy, blood samples	Creation of a repository of iPSCs from patients with spinal muscular atrophy as secondary objective	Others	February 2016	March 2022	1000	USA
21	FOXP1 Syndrome: The Seaver Autism Center for Research and Treatment is Characterizing FOXP1-related Neurodevelopmental Disorders Using Genetic, Medical, and Neuropsychological Measures.	NCT03718923	Blood samples	Creation of iPSCs from patients with FOXP1 syndrome as secondary objective		March 2016	March 2023	30	USA
22	The Crnic Institute Human Trisome Project Biobank (HTP)	NCT02864108	Blood samples	Creation of a repository of iPSCs from patients with Down's Syndrome		July 2016	July 2036	1500	USA
23	DDX3X Syndrome -The Seaver Autism Center for Research and Treatment is Characterizing DDX3X-related Neurodevelopmental Disorders Using Genetic, Medical, and Neuropsychological Measures.	NCT03718910	Blood samples	Creation of iPSCs from patients with DDX3X syndrome as secondary objective		May 2018	August 2024	10	USA
24	iPSC-based Drug Repurposing for ALS Medicine (iDReAM) Study	NCT04744532	Drug: Bosutinib	iPSC-based drug repurposing of bosutinib in patients with ALS		March 2019	March 2022	24	Japan

25	Diagnosis of RSTS: Identification of the Acetylation Profiles as Epigenetic Markers for Assessing Causality of CREBBP and EP300 Variants. (GENEPI)	NCT04122742	Skin biopsy	Induction of iPSCs from patients with Rubinstein-Taybi syndrome for histone acetylation profiling		October 2019	October 2022	154	France
26	Classification, Functional Stratification and Biomarkers in Ciliopathy (CILLICORIRCM)	NCT04874909	Blood samples	Induction of iPSCs from patients with ciliopathies		January 2021	January 2024	240	France
27	Development of iPS From Donated Somatic Cells of Patients With Neurological Diseases	NCT00874783	Skin biopsy, hair samples	Creation of iPSCs repository from patients with neurodegenerative disorders		April 2009	December 2025	120	Israel
28	Characterization of Patients With Uncommon Presentations and/or Uncommon Diseases Associated With the Cardiovascular System	NCT01143454	Blood samples	Induction of iPSCs from patients at risk for cardiovascular diseases as secondary objective		July 2010	N/A	5000	USA
29	Derivation of Induced Pluripotent Stem Cells From an Existing Collection of Human Somatic Cells	NCT00801333	Cells from existing collection	Creation of iPSCs repository from patients with ALS		November 2008	December 2025	25	Israel
30	Biomarkers for Pain in Spinal Cord Injury (SCI) Patients (SCI Pain)	NCT00913471	Skin biopsy, blood samples	Induction of iPSCs from patients with Traumatic Spinal Cord Injury as secondary objective		June 2009	December 2030	120	USA
